# Distinct Neural Substrates Support Phonological and Orthographic Working Memory: Implications for Theories of Working Memory

**DOI:** 10.3389/fneur.2021.681141

**Published:** 2021-08-04

**Authors:** Jeremy Purcell, Brenda Rapp, Randi C. Martin

**Affiliations:** ^1^Maryland Neuroimaging Center, University of Maryland, College Park, MD, United States; ^2^Cognitive Science Department, Johns Hopkins University, Baltimore, MD, United States; ^3^Department of Psychological Sciences, Rice University, Houston, TX, United States

**Keywords:** working memory, phonological working memory, orthographic working memory, multivariate lesion symptom mapping, working memory deficits, buffer theories, embedded processes theories

## Abstract

Prior behavioral and neuroimaging evidence supports a separation between working memory capacities in the phonological and orthographic domains. Although these data indicate distinct buffers for orthographic and phonological information, prior neural evidence does indicate that nearby left inferior parietal regions support both of these working memory capacities. Given that no study has directly compared their neural substrates based on data from the same individuals, it is possible that there is a common left inferior parietal region shared by both working memory capacities. In fact, those endorsing an embedded processes account of working memory might suggest that parietal involvement reflects a domain-general attentional system that directs attention to long-term memory representations in the two domains, implying that the same neural region supports the two capacities. Thus, in this work, a multivariate lesion-symptom mapping approach was used to assess the neural basis of phonological and orthographic working memory using behavioral and lesion data from the same set of 37 individuals. The results showed a separation of the neural substrates, with regions in the angular gyrus supporting orthographic working memory and with regions primarily in the supramarginal gyrus supporting phonological working memory. The results thus argue against the parietal involvement as supporting a domain-general attentional mechanism and support a domain-specific buffer account of working memory.

## Introduction

Studies of verbal working memory (WM) have often focused on phonological WM - the capacity for maintaining phonological codes for words or non-words as assessed by memory span (e.g., recall of a list of digits in order) or recognition/probe tasks (e.g., judging whether a probe word or non-word matches any item in a preceding list) (1–3). However, neuropsychological evidence has supported the existence of other verbal WM capacities, specifically those involved in maintaining semantic (4, 5) and orthographic information [for an overview see (6, 7)]. With regard to the latter, orthographic WM (also referred to as the graphemic buffer) is argued to be involved in retaining the identity and order of letters during the spelling of individual words and is assessed by the effects of word length on accuracy and the types of errors that are made in spelling [(8), and see (9), for related findings from healthy individuals]. Although some neuropsychological studies have provided evidence for a separation of capacities for phonological and orthographic WM, with patients having orthographic WM deficits performing well on tests of phonological WM (e.g., repetition of non-words of different lengths, digit span, etc.) (6, 10), for the most part, studies of phonological WM deficits have not assessed orthographic WM [e.g., (2, 11, 12)]. Furthermore, no studies have directly compared the lesion localizations associated with deficits in the two domains. The aim of the current study is to use lesion-symptom mapping on the same set of brain-damaged individuals to determine whether there is neural evidence for distinct phonological and orthographic WM capacities.

Whether one might predict distinct neural regions supporting the two WM capacities depends on the theoretical orientation that is adopted – that is, buffer [e.g., (13, 14)] vs. embedded processes approaches to WM (15, 16). Traditionally, models of WM assume domain-specific buffers for maintaining information over a short term. For instance, the well-known Baddeley WM model assumes a phonological loop and a visual-spatial sketchpad, which are specialized stores for maintaining speech-sound and visual-spatial representations, respectively (13, 17). These are assumed to be separate from Long Term Memory (LTM) for language or visual-spatial representations. In contrast, recent embedded processes approaches reject the notion of specialized WM stores, instead positing that WM consists of the activated portion of LTM – that is, the set of LTM representations recently activated due to processing environmental input or due to internal thought processes (15). Within the set of activated representations, a small number of items [e.g., from one to four according to different models - (18, 19)] is assumed to be held within the focus of attention, with these items potentially coming from different domains (e.g., verbal and visual-spatial simultaneously).

**Figure 1:**
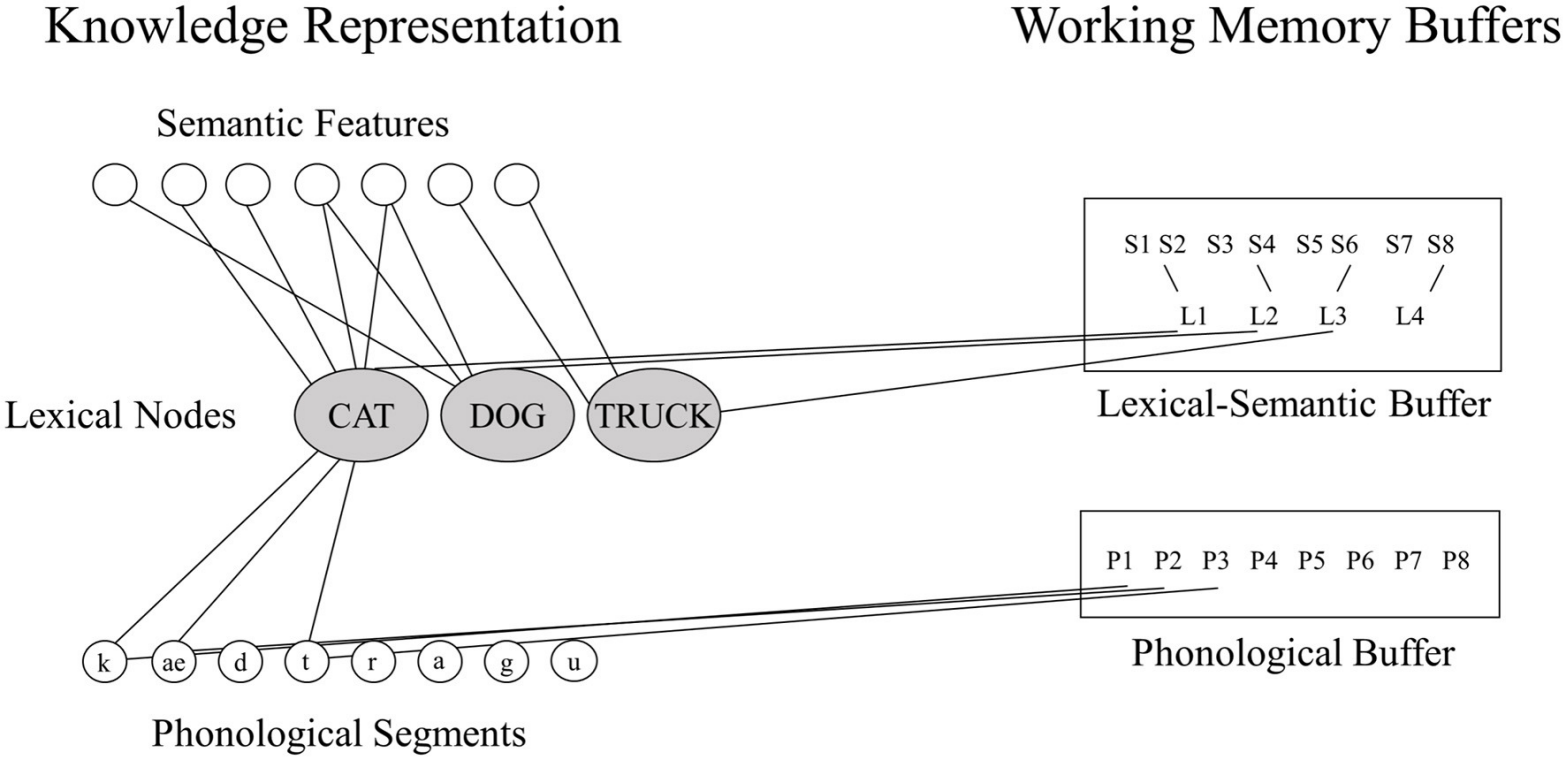
The domain-specific model of phonological and semantic WM [adapted from (14)].

Neuropsychological researchers have often favored the buffer approach because of striking dissociations between WM and LTM in a given domain (7). For example, in the phonological domain, individuals with very reduced phonological WM capacity as measured by digit or word span (e.g., digit spans of 1 or 2 items compared to 5–7 digits for controls) have been shown to have preserved single word comprehension and production (2, 3, 12), suggesting that their speech perception and long-term representations for phonological forms (phonemes, syllables, and words) are preserved. Martin and colleagues (14) have put forward a model of working memory in which there is a tight linkage between LTM representations for lexical items and working memory storage, but which includes separable storage buffers that may be independently affected by brain damage (see Figure 1) (In this model, there is a separation between buffers for maintaining phonological and semantic information; however, the current study will focus on the phonological WM buffer.). In contrast to the buffer approach, some neuropsychological researchers studying phonological WM have advocated theoretical positions similar to the embedded processes approach, arguing that subtle phonological processing problems, which can be detected with sufficiently demanding tasks, are actually the source of phonological WM deficits (11, 20). However, there is evidence that such subtle phonological impairments cannot readily account for severely restricted phonological WM capacity (7, 21).

**Figure 2:**
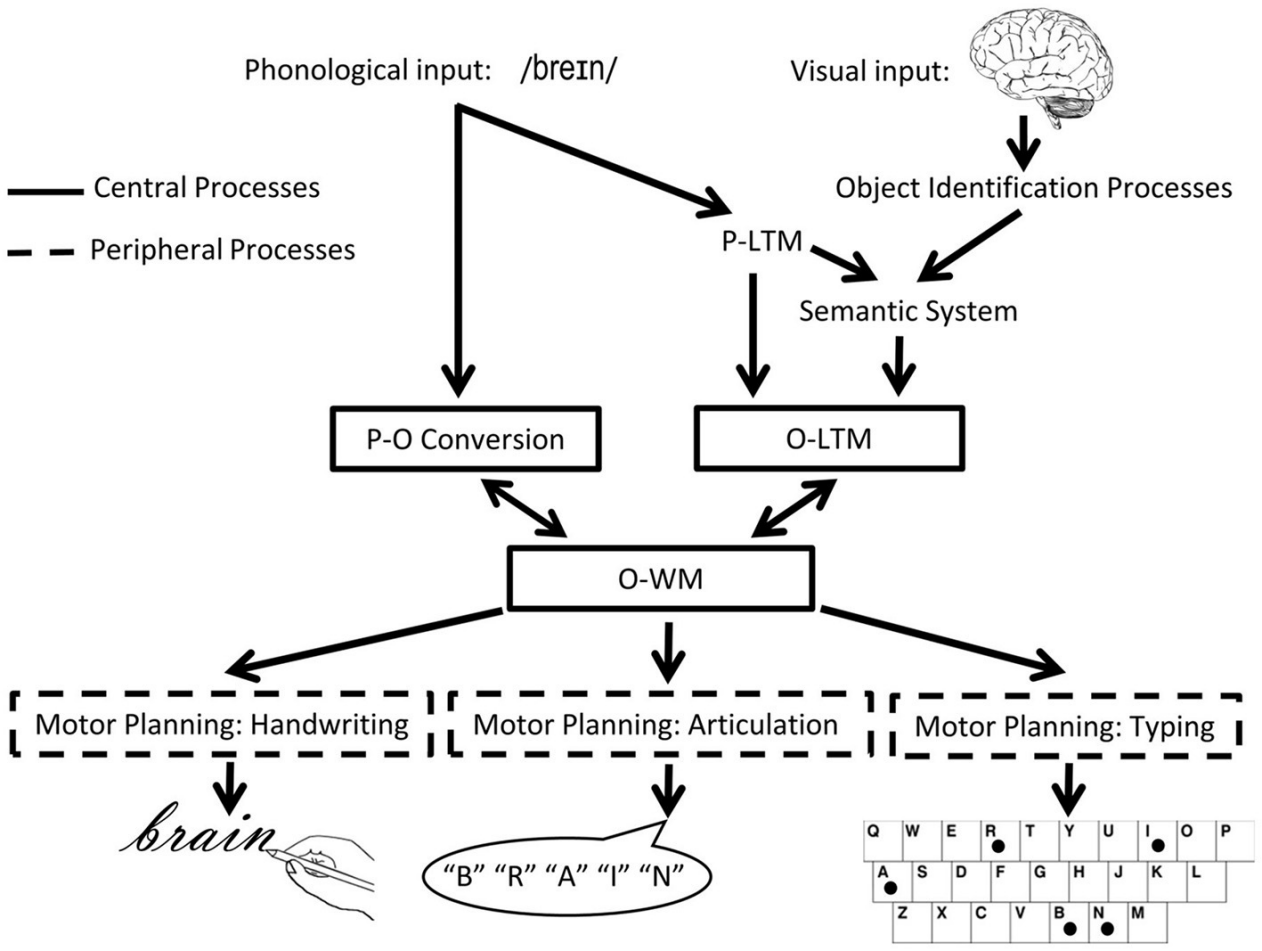
The cognitive architecture of spelling. Schematic depiction of the cognitive processes involved in spelling, highlighting central (core) and peripheral processes. P, phonological/phonology; O, orthographic/orthography; WM, working memory [adapted from (22)].

In the orthographic domain, there is also evidence for a separation between WM and LTM. Consider the model of spelling shown in Figure 2. According to this model, spellings of familiar words are retrieved from orthographic LTM memory in response to a dictated word or the concept of a word to be communicated. Also, plausible spellings of unfamiliar words or pseudowords can be generated via the application of phoneme-to-grapheme conversion processes. However, whether generated by the lexical or sublexical routines, the strings of abstract letter representations and their order are processed by orthographic WM. Orthographic WM is responsible for maintaining orthographic information in an active state while the serial selection of each letter takes place so that the information can be produced in a specific format for written spelling, oral spelling, typing etc. The independence of Orthographic LTM and WM systems is supported by the finding of distinct patterns of performance subsequent to brain damage (10). Disruption to orthographic LTM has been associated with the following pattern: sensitivity to the frequency of a word, with more errors for low compared to high frequency words, insensitivity to the length (in letters) of the word, typically accompanied by word-level errors (phonologically plausible errors or lexical substitutions). Disruption to orthographic WM has been associated with the contrasting pattern: insensitivity to word frequency but sensitivity to word length, with higher rates of errors for letters in longer compared to shorter words, and letter level errors (letter deletions, substitutions, and transpositions). While most theories of spelling posit orthographic LTM and orthographic WM processes, there has been debate regarding the independence of these processes and, more specifically, regarding the degree to which they can be independently disrupted (23–26).

In addition to this evidence of the separation of WM and LTM in both phonological and orthographic domains, neural evidence suggests that there are distinct neural substrates underlying each WM domain (see Table 1). In the phonological domain, a number of findings from different methodologies implicate the left supramarginal gyrus (SMG) in phonological WM: (1) lesion overlap (29); (2) multivariate lesion symptom mapping (5, 30) (3) univariate and multivariate analyses of fMRI data from healthy individuals (27, 31, 32). In contrast, speech perception and the representation of phonemes, syllables and words involves the left superior temporal gyrus and left superior temporal sulcus (27, 32–34). In the orthographic domain, lesion symptom mapping has revealed an inferior parietal region as supporting the graphemic buffer and posterior inferior frontal and inferior temporal-occipital regions supporting orthographic LTM. Neuroimaging results converge with these findings. Rapp and Dufor (28) reported findings from an fMRI study that examined BOLD response to written spelling of words that varied in length (long vs. short) and frequency (high vs. low). They found brain areas sensitive to word length but not frequency in the left posterior parietal lobe and the superior frontal gyrus, and they found the reverse pattern of sensitivity to word frequency but not length in the left inferior frontal lobe and the left fusiform gyrus. For additional convergent findings see also (35, 36).

**Table 1:** Centroids of brain regions associated with phonological and orthographic WM from prior studies using lesion symptom mapping or functional MRI of healthy individuals.

	**Phonological WM**	**Orthographic WM**
Lesion symptom mapping	−52 −30 38^a^	−39 −50 36^c^
fMRI of healthy	−53 −35 24^b^	−28 −50 52^d^

a *Martin et al. (5)*.

b *Yue et al. (27)*.

c *Rapp et al. (10)*.

d *Rapp and Dufor (28)*.

An important question remains, however, regarding whether the two parietal regions involved in phonological and orthographic WM can be differentiated within the same study. In alphabetic writing systems, there is a close association between phonological and orthographic codes and the same mechanism has been postulated to represent both the order of speech sounds and orthographic codes in WM (37), lending credence to the possibility that perhaps a single WM system supports maintenance in the two domains. Moreover, as discussed earlier, embedded processes approaches assume an attentional spotlight that holds information in the focus of attention, which can be directed toward representations in different LTM stores (15). A large body of research implicates parietal regions in attentional systems (38). Thus, one might postulate that those with phonological or orthographic WM deficits have a disruption of the attentional spotlight component of WM which directs attention to phonological or orthographic LTM representations. At odds with this proposal however, are prior lesion and fMRI data suggesting that the localization of the orthographic buffer is more medial and posterior than the phonological buffer (see coordinates in Table 1). Nonetheless, the centroids are fairly close and the lesion symptom mapping study of orthographic WM and orthographic LTM of Rapp and colleagues (10) uncovered a large parietal region supporting orthographic WM which extended into the SMG. Stronger evidence for a separation of the two regions would be obtained from a comparison of the involvement of regions supporting phonological WM and orthographic WM in the same study with the same individuals. Thus, in the current study, we evaluated whether the neural substrates for phonological and orthographic WM differ, as revealed through multivariate lesion symptom mapping of individuals with brain damage who were assessed on phonological WM and orthographic WM capacity. If distinct regions can be identified, such findings would argue against claims that the parietal involvement in both WM capacities is due to the engagement of a domain-general attentional mechanism.

## Methods

### Participants

Thirty-seven individuals (23/14 Male/Female) were included based on the following criteria: a left hemisphere lesion (35 strokes and 2 tumor resections; 1 had an additional, separate right hemisphere stroke), have an acquired impairment in spelling and/or phonological working memory, no contraindication for MRI, and no other neurological disease. Enrollment occurred across two sites; thirty-three participants were enrolled from Johns Hopkins University and four participants were enrolled from Rice University. Each individual participated in behavioral testing and structural MRI scanning. See Table 2 for demographic, lesion and other information. Consent was obtained using procedures consistent with the Declaration of Helsinki and the Johns Hopkins University or Rice University Institutional Review Boards.

**Table 2:** Participant characteristics and lesion details.

**ID**	**Age (yrs)**	**Gender**	**Hand**	**Ed (yrs)**	**Etiology**	**Lesion location**	**Lesion volume (cc)**	**Post-onset (months)**
ABS	58	M	R	18	stroke	Left F	162.16	97
AEF	55	F	R	16	stroke	Left F/P	249.73	101
AES	59	F	L+R	16	stroke	Left F/P	210.68	209
BWN	87.4	M	R	18	stroke 1	Left P	85.24	179
					stroke 2	Right P	16.003	155
CIE	62	F	L	14	stroke	Left F	53.89	85
CSS	63.5	M	R	15	stroke	Left F/P	60.6	50
DBY	54.3	F	R	14	stroke	Left O/T	4.24	44
DHY	37.3	M	R	16	stroke	Left F/P	111.96	35
DPT	36	M	R	19	tumor	Left O/T	22.91	48
DSK	67	M	R	16	stroke	Left F/P	158.57	59
DSN	68.8	F	R	16	tumor	Left O/T	20.95	26
DTE	80	F	R	18	stroke	Left F/P	75.79	14
ESG	62	M	L	16	stroke	Left F/P	120.64	38
FCE	64	M	R	12	stroke	Left F	50.58	119
JGL	72	F	R	16	stroke	Left O/T	41.57	32
JRE	75	F	R	18	stroke	Left F/P	103.39	207
KMN	55	M	R	15	stroke	Left F/P	61.01	28
KST	61	M	L+R	14	stroke	Left F	21.72	46
LC	67	M	NA	13	stroke	Left P/T	144.42	159
LHD	71.9	F	R	18	stroke	Left O/T	70.36	70
LHT	74	M	R	16	stroke	Left F/P/T	92.97	165
LPO	42.3	F	R	18	stroke	Left P Lobe	66.18	29
LSS	54.1	M	L	18	stroke	Left Posterior F	51.44	3
MK	46	M	L	14.5	stroke	Left P/T	133.56	20
MLB	56	M	R	14	stroke	Left P/T	51.25	69
MSO	45	M	R	18	stroke	Left F/T	192.85	103
PP	66	M	L	20	stroke	Left P/T	15.02	40
PQS	54	M	R	18	stroke	Left F/P	106.42	17
RFZ	60	M	R	18	stroke	Left P/T	63.26	46
RHH	45	M	R	16	stroke	Left F/P	128.37	82
RHN	75	F	L	19	stroke	Left Posterior F	9.18	27
RSB	66.1	M	R	18	stroke	Left P	66.79	146
SDA	69	F	R	20	stroke	Left P/T	50.05	25
TCI	69	F	R	12	stroke	Left F/P	79.48	45
TCK	69	M	R	16	stroke	Left F	25.01	68
VBR	56.7	F	R	12	stroke	Left F/P	107.58	63
WCR	64	M	R	18	stroke	Left F/P/O/T	280.71	86

*ID, Participant Identification; M, Male; F, Female; R, right; L, Left; yrs, years; cc, cubic centimeters; T1, T1-Weighted structural scan; Flair, Fluid-attenuated inversion recovery T2-weighted structural scan; tumor, tumor resection; F, Frontal Lobe; P, Parietal Lobe; T, Temporal Lobe; O, Occipital Lobe; NA, not available*.

### Cognitive/Language Assessments

#### Phonological Working Memory

Digit forward span performance from the WMS-III (39) was used to determine phonological working memory capacity. The task was administered using the standard procedure from the WMS in which subjects are asked to repeat back two lists of digits at list lengths 2–9, stopping the administration when both lists are missed at a given length. Digit span was calculated as the longest list length at which both lists were repeated correctly plus 0.5 for each list correct at longer list lengths. For example, if an individual repeated correctly two lists at list length 2, one list at list length 3, and none at list length 4, digit span would be 2.5.

#### Phonological Long Term Memory

The Peabody Picture Vocabulary Test (PPVT) (40) was used to quantify phonological long-term (input) memory of word forms. These scores were included in the analysis to assist in identifying the neural substrates of phonological working memory while controlling for phonological long-term memory processing/representation.

#### Orthographic Working Memory

Spelling performance was measured using the JHU Dysgraphia Battery Length List (41) in all but 1 participant who instead received the Snodgrass word lists (42, 43). Letter accuracy (e.g., COAT as COET has a 75% letter accuracy, a misspelling as CUAD has a 50% letter accuracy) was used throughout rather than word accuracy, because it is both a more precise measure of spelling performance and more appropriate for quantifying orthographic working memory (8). Eight participants were tested twice on the JHU Length List within 6 months; the data from these two tests were averaged for these individuals. The OWM measure was defined categorically to indicate if an individual had a selective OWM impairment but not an Orthographic Long-Term Memory (OLTM) impairment (10). To obtain this measure, participants were first identified as having either an OWM and/or OLTM deficit in spelling. An OLTM deficit was defined by spelling performance that exhibited sensitivity to word frequency and/or the production of phonologically plausible errors. In other words, an OLTM deficit was identified when performance was significantly worse for low frequency (<15 per million) words relative to high frequency (>60 per million) words. An OWM deficit was defined as a sensitivity to word length (10) such that performance was significantly worse for long (7 and 8 letter) words relative to short (4 and 5 letter) words. On that basis, a categorical value (0 or 1) was assigned to each participant based on OWM or OLTM deficit status.

### Structural MRI: Data Acquisition and Lesion Tracing

All scans were whole brain imaging and were acquired on 3 Tesla MRI scanners at F.M. Kirby Center for Functional Brain Imaging at the Kennedy Krieger Institute (Baltimore, MD) or the Center for Advanced Magnetic Resonance Imaging (CAMRI) at Baylor College of Medicine, except for one which was acquired on a CT scanner. Whereas, two MRI structural scans were acquired at a resolution of 1 × 1 × 5 mm, the rest were acquired with a resolution of 1 × 1 × 1 mm. Twenty-six of the participants with T1-weghted images also had Fluid Attenuated Inversion Recovery (FLAIR) scans.

**Figure 3:**
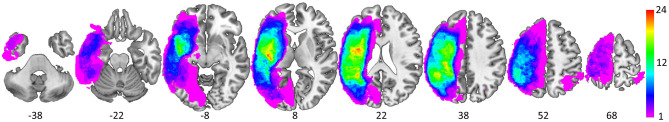
Spatial distribution of the 37 lesions as depicted via equally spaced (16 mm) axial slices (MNI z coordinate below each slice). The color scale denotes the number of overlapping lesions at each voxel.

Each structural scan was aligned to the AC-PC plane and resampled to 1 × 1 × 1 mm isotropic voxels. All lesion masks were drawn using MRIcron (44). First, lesion masks were drawn in hypointense lesion voxels in either the T1-weighted or the CT scan. Second, for those with FLAIR scans, additional lesion drawing was performed on hyperintense voxels in the same hemisphere as the already drawn lesion (45, 46). Given that our participants were primarily older, images were normalized to a standard older T1 weighted template (47). In order to account for brain lesion abnormalities, the lesion space was filled with estimated intact tissue from the contralesional hemisphere prior to normalization as per the enantiomorphic normalization approach using SPM12 in MATLAB (48). Normalization parameters were applied to the structural and functional data for normalization to MNI space. Analyses were constrained to a gray matter mask [Harvard-Oxford atlas cortical and sub-cortical regions (49)]; see Figure 3 for a lesion distribution map and Table 3 for a brief description of the lesion location for each participant.

**Table 3:** Behavioral profiles and indices of PWM, OWM, PLTM and OLTM used in the analyses.

			**Spelling performance**			
**ID**	**PPVT %ile**	**Digit span**	**Freq effect Chi-square *p*-value**	**Presence of PPEs**	**Length effect Chi-square *p*-value**	**Type of spelling deficit**
ABS	61	5.5	0.002	Yes	0.084	OWM + OLTM
AEF	1	3.5	0.001	Yes	0.031	OWM + OLTM
AES	39	5.5	<0.001	Yes	0.633	OLTM
BWN	94	2.5	0.870	No	0.010	OWM
CIE	1	4.0	<0.001	No	0.895	OLTM
CSS	42	4.0	0.130	No	<0.001	OWM
DBY	99	4.5	0.576	Yes	0.071	OLTM
DHY	87	5.0	0.002	Yes	0.667	OLTM
DPT	92	9.0	0.159	Yes	0.711	OLTM
DSK	55	3.5	<0.001	Yes	0.019	OWM + OLTM
DSN	91	5.5	0.015	Yes	0.235	OLTM
DTE	91	5.0	0.154	No	<0.001	OWM
ESG	6	4.0	0.036	Yes	0.017	OWM + OLTM
FCE	23	5.0	<0.001	Yes	<0.001	OWM + OLTM
JGL	60	6.0	0.061	Yes	0.300	OLTM
JRE	94	5.0	0.709	No	<0.001	OWM
KMN	27	0.0	<0.001	Yes	0.058	OWM + OLTM
KST	10	3.0	<0.001	Yes	0.004	OWM + OLTM
LC	97	3.0	0.024	Yes	0.671	OLTM
LHD	55	8.0	0.100	Yes	0.165	OLTM
LHT	1	3.0	0.245	No	0.920	OLTM
LPO	73	4.5	0.693	No	0.021	OWM
LSS	7	5.0	<0.001	No	0.450	OLTM
MK	4	3.5	1.000	Yes	0.021	OLTM
MLB	47	3.5	0.096	Yes	0.319	OLTM
MSO	50	3.5	0.002	No	<0.001	OWM + OLTM
PP	77	3.5	0.009	Yes	0.356	OLTM
PQS	99	5.0	0.184	Yes	<0.001	OWM + OLTM
RFZ	79	4.5	0.325	Yes	<0.001	OLTM
RHH	87	4.5	0.317	Yes	0.438	OLTM
RHN	73	6.0	0.007	Yes	0.027	OLTM
RSB	97	4.0	0.406	No	0.002	OWM
SDA	73	3.5	0.008	Yes	0.468	OLTM
TCI	30	4.5	0.001	Yes	0.126	OLTM
TCK	50	3.5	<0.001	Yes	0.002	OWM + OLTM
VBR	58	5.0	<0.001	Yes	0.271	OLTM
WCR	84	4.0	<0.001	Yes	<0.001	OWM + OLTM

*ID, Participant Identification; PPVT, Peabody Picture Vocabulary Test; Acc., Accuracy; Freq, Frequency; PPE, phonologically plausible errors (e.g., BOTE for BOAT). High Frequency = >60 per million; Low Frequency = <15 per million; Short = 4 and 5 letter words; Long = 7 and 8 letter words. Spelling Deficits were classified as either an OWM - Orthographic Working Memory or OLTM - Orthographic Long Term Memory deficit. An OWM deficit = a Sig/Trend for length effect. An OLTM deficit = a Sig/Trend for frequency effects OR the presence of PPEs*.

### Multivariate Lesion Symptom Mapping

In order to localize brain regions that are associated with phonological and orthographic working memory, support vector regression LSM (SVR-LSM) was used (50, 51). SVR-LSM considers the lesion status of all voxels in a single regression model, and is not only sensitive to non-linear relationships but can identify multiple brain regions supporting a cognitive function of interest (51). We applied SVR-LSM *via* a MATLAB toolbox (50). Only voxels lesioned in at least four participants (~10%) were included in the analyses. Two analyses were carried out - one for Phonological Working Memory and the other for Orthographic Working Memory, each designed to specifically identify neural substrates of working memory while accounting for the integrity of domain-specific (phonological or orthographic) long-term memory. Whereas PWM deficits can be directly quantified by the magnitude of the *continuous variable* digit span [e.g., (30)], OWM deficits are less directly quantified with a single variable given such deficits need to be distinguished from impairments affecting other functions of the central spelling system (Figure 2), OLTM in particular. Given there are no clear continuous variables differentiating OWM from OLTM impairments, it was necessary to use a *categorical variable* to identify selective OWM deficits. Specific details are provided below.

#### Phonological Working Memory Analysis

Phonological working memory performance was defined using the digit span for each participant. This measure corresponded to the dependent variable in the SVR-LSM. This analysis also included a covariate indexing performance on the PPVT (see above) that was used to control for phonological long-term memory performance.

#### Orthographic Working Memory Analysis

Orthographic working memory deficits were defined by the presence of an OWM deficit but not an OLTM deficit. For each participant with a selective OWM deficit this variable was set to −1 (6 in total) while those with a selective OLTM deficit were set to 1 (20 in total). Next, this variable was demeaned, and then for the eleven who had both an OWM and an OLTM deficit (11 in total) the value was set to 0. In this way, substrates selectively associated with OWM could be identified.

Three additional covariates of non-interest were used in both analyses. Both age and gender were included to account for any systematic relationships to age-related variability in performance and gender-related differences in language localization in the brain (52). Both of these have been used in previous working memory VLSM studies (53, 54). The third covariate was lesion volume that is standardly used in order to avoid the possibility that the locations of VLSM results could be driven by the tendency of large lesions to overlap, spuriously identifying overlapping lesioned voxels as being related to a cognitive process of interest (50). All covariates were z-normalized relative to the sample (i.e., each was demeaned and then divided by the group standard deviation).

Voxel-based significance was performed separately for each analysis and was determined using a continuous permutation-based FWER approach (55). Briefly, the behavioral data was permuted 5,000 times and the SVR-LSM VLSM analyses were run on each of these random data sets. The maximum voxel-wise β value was identified for each of 5,000 iterations to build a null distribution of maximum β values that were observed by chance. For each voxel, the threshold of *p* = 0.05 was then set relative to this null distribution of maximum β values obtained by chance. To evaluate the findings at more lenient thresholds, null distributions were also generated based on the 10th, 100th, and 1,000th highest β values observed in the 5,000 random permutations. Cluster locations were defined using the Harvard Oxford Cortical Atlas (49).

## Results

**Figure 4:**
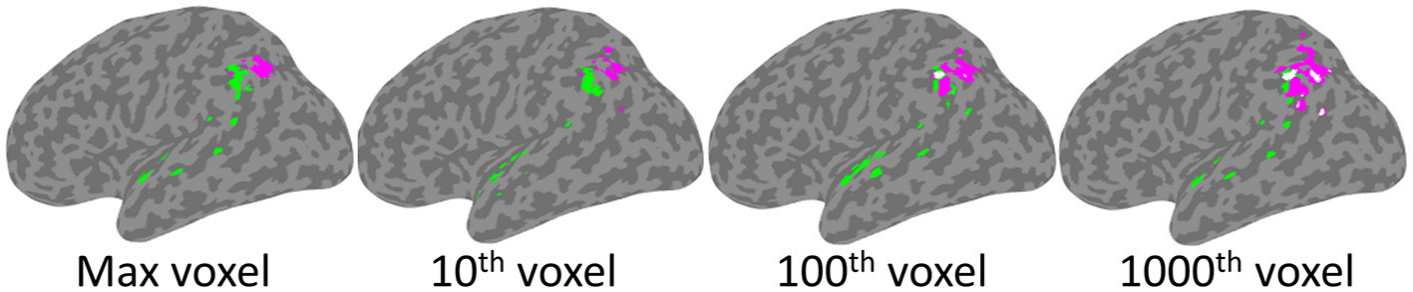
SVR-LSM results for Phonological Working Memory (green) and Orthographic Working Memory (magenta). Clusters projected onto a left hemisphere standard MNI152 cortical surface using mni2fs (56). Each image depicts the results using thresholds which, from left to right, are based on increasingly more lenient thresholds from the permutation analysis. Although there is some overlap (white) at the two most lenient thresholds, there is none at the more conservative max and 10th voxel thresholds.

As depicted in Figure 4, significant clusters were observed for the Phonological Working Memory (PWM) analysis in the temporal-parietal cortex and for the Orthographic Working Memory (OWM) analysis in the posterior parietal cortex. These were observed using the threshold based on maximum chance values, as well as for the more lenient thresholds. Interestingly, for the more conservative thresholds (based on maximum voxel and 10th highest) there was no overlap between the PWM and the OWM clusters. Only at the more liberal threshold (100th and 1,000th highest voxel) was there any overlap (white clusters in Figure 4). This reveals that although the PWM and OWM substrates are adjacent to one another within the left parietal cortex, even at the most lenient thresholds there were clearly non-overlapping voxels.

As presented in Table 4, PWM clusters based on the most stringent threshold were distributed across left tempo-parietal cortex, where they were specifically concentrated within the left supramarginal gyrus. Additional large clusters were observed in the parietal operculum and the superior temporal gyrus. There were also relatively small clusters observed in the planum polare and angular gyrus. On the other hand, OWM was only associated with the angular gyrus, in a region just inferior and adjacent to the intraparietal sulcus.

**Table 4:** Left hemisphere SVR-LSM results: clusters of significant voxels that surpass the most stringent *voxel* thresholding based on a distribution of maximum beta values obtained from analyses of 5,000 random permutations of the data set.

**Location**	**Volume (mm^**3**^)**
**Phonological working memory**	
Supramarginal gyrus (posterior)	103
Supramarginal gyrus (posterior)	16
Supramarginal gyrus (posterior)	14
Angular gyrus	11
Parietal operculum^†^	99
Superior temporal gyrus (posterior)	58
Planum polare	18
**Orthographic working memory**	
Angular gyrus	501

† *centroid outside of gray matter, this is the nearest location (within 2 mm)*.

*These results are visualized in the left-most image in Figure 4*.

## Discussion

This study addressed the question of whether different neural regions support phonological and orthographic WM. If so, the findings would converge with behavioral findings implying separable WM capacities in the two domains. The results of the multivariate LSM analysis of individuals assessed on both capacities revealed that, to a large extent, the regions supporting the two capacities were distinct. Overlap was only observed when adopting the most liberal significance thresholds and even at those thresholds, there were many non-overlapping voxels. In line with prior functional neuroimaging and LSM evidence obtained from studies examining either phonological (5, 27, 31) or orthographic WM (10, 28), the largest region associated with phonological WM was in the SMG, more anterior and lateral than that for orthographic WM, where one large region in the angular gyrus was observed. Given that mainly distinct regions were identified for the two capacities, the findings argue against the notion associated with the embedded processes view that the left inferior parietal region supporting these WM capacities is the neural substrate for a common attentional mechanism.

Embedded processes theorists might counter that distinct attentional mechanisms are instantiated in these parietal regions, with one involved in maintaining orthographic information and the other in maintaining phonological information in the focus of attention, consistent with some proposals for domain-specific attentional capacities (57). A strong argument against assuming that these parietal regions support attentional mechanisms comes from multivariate imaging analyses which have provided evidence that phonological information can be decoded in the SMG during a delay period in a phonological WM task (27, 32). One would not expect such decoding from a region instantiating attentional processes. Of course, it would be valuable to extend this research to show that orthographic codes could not be decoded in the SMG but could in the AG during WM maintenance. However, in the orthographic domain, deficits arising from lesions to these areas produce errors that reflect the properties of orthographic representations. For example, lesions to the left parietal cortex (8) often result in a pattern of spelling errors such that orthographically (and not phonologically) defined consonants are substituted for consonants and vowels for vowels, a pattern not predicted by an attentional account.

Another prediction associated with the embedded processes view is that the same regions should support WM and LTM in a given domain, as WM consists of the activated portion of LTM [e.g., (15)]. In the orthographic domain, however, the angular gyrus region revealed here and in prior studies is distinct from the inferior temporal-occipital and inferior frontal regions previously found to be associated with orthographic LTM (10, 28, 36). In the phonological domain, the SMG region, in which the largest number of significant voxels was observed, is distinct from superior temporal regions typically thought to support speech perception and representations of phonemes, syllables, and phonological word forms (27, 32–34). Nonetheless, in the current study, for phonological WM, significant regions were also identified in the posterior superior temporal gyrus and planum polare. These results contrast with those from a recent multivariate VLSM study examining phonological and semantic WM in individuals at the acute stage of stroke (5), in which no superior temporal or primary auditory areas were associated with phonological WM [see also, (32), for related neuroimaging findings]. Several factors might explain the discrepancy. For one, it is possible that some reorganization of function has occurred for the individuals studied here who were, on average 6.2 years post-stroke, with temporal regions taking over WM functions to some extent. Second, it is possible that our measure of phonological processing and phonological LTM [picture-word matching from the PPVT, (40)] was not a sufficiently stringent measure of phonological processing. That is, while speech perception and lexical access are required for this task, the materials do not include distractor pictures with phonologically related names, in contrast to the picture-word matching task used with the acute stroke patients in Martin et al. (5). Thus, it is possible that the individuals included here had speech perception deficits that affected their performance on the phonological WM task. On the other hand, it should be acknowledged that coverage in the superior temporal region was low in the Martin et al. (5) study, making it difficult to identify regions critical to phonological WM in this region. Thus, this previous study may have missed a potential contribution from these superior temporal lobe regions. Nonetheless, the Martin et al. (5) study did indicate that damage to these superior temporal regions was not necessary for a phonological WM deficit, as patients without damage to those regions showed substantial phonological WM deficits.

In general, the current findings provide support for a buffer account of WM, with separate buffers supporting phonological and orthographic WM. One complaint lodged against a buffer approach is that it seems unconstrained, opening up the possibility for a multitude of different buffers for maintaining different types of information. In contrast, the embedded processes approach might be seen as more parsimonious, as differential disruption to WM in various domains can be attributed to a disruption of perception or LTM in a given domain, where there is an existing consensus that different brain regions support LTM representations in different domains (58, 59). However, as discussed in Martin et al. (7), while we allow for the possibility of multiple buffers, we do not suggest that they exist for all types of information. We endorse the proposal of Caramazza et al. (6) that buffers are computationally motivated when there is a mismatch between the size of units at the interface between two processing levels. For example, in spelling, letters and their positions for a specific word may be retrieved simultaneously from orthographic LTM, but then a buffer is needed to maintain those representations in WM while the serial spelling process takes place. In the other direction, during comprehension, one hears a sequence of speech sounds and a buffer is needed to maintain the multiple speech sounds until words are identified. Although a sequential elimination process for word candidates based on incoming individual phonemes has sometimes been suggested, eliminating the need for a phonological buffer [e.g., in the original cohort model (60)], it is often the case that word boundaries may be ambiguous until subsequent information is processed (as in “I *bet her* five dollars” vs. “I *better* do my laundry,” where the underlined information may be pronounced identically), requiring that phonological inputs be available until sufficient disambiguating information has been presented. It is also possible that a sequence of phonemes represents an unfamiliar proper name or other novel word and a buffer is needed to maintain and identify the boundaries of the novel sequence and bind these segments together to create new word representations (61, 62).

If one adopts the position advocated by Caramazza et al. (6), then it follows that maintenance over the short term will involve a buffer when it is part of a processing system where this transition between different sizes of units is required. In contrast, for many types of information where it is hard to imagine what the different size units are and why such an interface might be needed, maintenance over the short-term would not depend on a buffer and may well depend on a system like that proposed by an embedded processes account. For instance, consider memory for a random sequence of faces, where memory is tested with a probe item or an n-back procedure [e.g., (63)]. In contrast to ordered lists of letters, phonemes or words, there is no naturally occurring unit to encompass a sequence of faces. Performance in these instances may depend on the formation of novel long-term memory representations of the faces themselves if unfamiliar and the binding of these representations to a temporal/spatial context for both familiar and unfamiliar faces. Persisting activation of these novel LTM representations would support performance in the task, in line with an embedded processes account. If this view is correct, then neuroimaging or lesion studies of WM in these domains would show evidence of the involvement of LTM for these domains (e.g., fusiform face area). Given this logic, there may be many domains where an embedded processes approach holds and a more circumscribed set where a buffer is involved. Of course, future work would be needed to determine a principled set of criteria for predicting where a buffer is required and the development of tests to determine if these predictions hold regarding the separation or identity of WM and LTM processes and their neural instantiations.

### Potential Weaknesses

One potential weakness is that the lesion locations in this study were limited in their coverage to roughly two thirds of the left hemisphere voxels (i.e., voxels lesioned in at least 4 individuals were included in the VLSM analysis). This weakness—shared by nearly every LSM study to date—means one cannot make claims about brain regions that are consistently intact. Thus, although our findings do provide support for the distinct phonological and orthographic WM buffers, they do not preclude the possibility that there is still another region that serves as a domain general WM function necessary for both buffers (e.g., in the right hemisphere which is completely excluded from this analysis).

Another possible weakness is the sample size of 37 which is in the small range for VLSM [e.g., 20–40 as discussed in (55)]. Mitigating this concern are the following points. First, given the small sample size in this study, we employed stringent statistical methods. That is to say, the relatively common False Discovery Rate (FDR) correction-for-multiple-comparisons approach is not appropriate for small sample sizes in VLSM (55); for this reason we used the more appropriate and stringent Family Wise Error (FWE) approach. Second, although the sample size was small, it is well within the range recent published VSLM studies [e.g., (64, 65)].

### Conclusions

The current study used multivariate lesion-symptom mapping to identify separate parietal cortex regions critical for phonological WM and orthographic WM. Whereas phonological WM was primarily associated with the left supramarginal gyrus, orthographic WM was associated with the more posterior left angular gyrus. Further, these domain specific WM capacities were distinct from their respective long-term memory (LTM) domains, i.e., phonological and orthographic LTM. These findings argue against the notion that the left inferior parietal cortex serves a domain general working memory function as suggested by the embedded processes accounts and instead provides support for a domain specific phonological and orthographic buffer account.

## Data Availability Statement

The neuroimaging data supporting the conclusions of this article are available at the following website: https://github.com/jpurcel8/Distinct-Neural-Substrates-Support-P-and-O-WM_Figure3-4.

## Ethics Statement

The studies involving human participants were reviewed and approved by Johns Hopkins University Institutional Review Boards and Rice University Institutional Review Boards. The patients/participants provided their written informed consent to participate in this study.

## Author Contributions

RM, BR, and JP contributed to the experimental rationale, interpretation, and manuscript preparation. JP was primarily responsible for data analysis. RM and BR were primarily responsible for the theoretical framing. All authors contributed to the article and approved the submitted version.

## Conflict of Interest

The authors declare that the research was conducted in the absence of any commercial or financial relationships that could be construed as a potential conflict of interest.

## Publisher's Note

All claims expressed in this article are solely those of the authors and do not necessarily represent those of their affiliated organizations, or those of the publisher, the editors and the reviewers. Any product that may be evaluated in this article, or claim that may be made by its manufacturer, is not guaranteed or endorsed by the publisher.

## References

[1] Burgess N Hitch GJ. A revised model of short-term memory and long-term learning of verbal sequences. J Mem Lang. (2006) 55:627–52. 10.1016/j.jml.2006.08.005

[2] Vallar G Baddeley AD. Phonological short-term store, phonological processing and sentence comprehension: a neuropsychological case study. Cogn Neuropsychol. (1984) 1:121–41. 10.1080/02643298408252018

[3] Warrington EK Shallice T. The selective impairment of auditory verbal short-term memory. Brain J Neurol. (1969) 92:885–96. 10.1093/brain/92.4.885 5364015

[4] Martin RC. The critical role of semantic working memory in language comprehension and production. Curr Dir Psychol Sci. (2021). 10.1177/0963721421995178 PMC8594863 34789966

[5] Martin RC Ding J Hamilton AC Schnur TT. Working memory capacities neurally dissociate: evidence from acute stroke. Cereb Cortex Commun. (2021) 2:tgab005. 10.1093/texcom/tgab005 33870195 PMC8030664

[6] Caramazza A Miceli G Villa G Romani C. The role of the Graphemic Buffer in spelling: evidence from a case of acquired dysgraphia. Cognition. (1987) 26:59–85. 10.1016/0010-0277(87)90014-X 3608396

[7] Martin RC Rapp B Purcell J. Domain-specific working memory: Perspectives from cognitive neuropsychology. In: Logie R Camos V Cowan N editors. Working Memory: The State of the Science. Oxford, UK: Oxford University Press (2020). p. 235–81.

[8] Buchwald A Rapp B. Distinctions between orthographic long-term memory and working memory. Cogn Neuropsychol. (2009) 26:724–51. 10.1080/02643291003707332 20425660 PMC3145833

[9] Wing AM Baddeley A. Spelling errors in handwriting: a corpus and a distributional analysis. In: Frith U editor. Cognitive Processes in Spelling. Academic Press (1980). p. 251–85.

[10] Rapp B Purcell JJ Hillis AE Capasso R Miceli M. Neural bases of orthographic long-term memory and working memory in dysgraphia. Brain. (2016) 139:588–604. 10.1093/brain/awv348 26685156 PMC4805091

[11] Belleville S Caza N Peretz I. A neuropsychological argument for a processing view of memory. J Mem Lang. (2003) 48:686–703. 10.1016/S0749-596X(02)00532-6

[12] Martin RC Shelton JR Yaffee LS. Language processing and working memory: neuropsychological evidence for separate phonological and semantic capacities. J Mem Lang. (1994) 33:83–111. 10.1006/jmla.1994.1005

[13] Baddeley AD Hitch GJ Allen A. A multicomponent model of working memory. In: Logie R Camos V Cowan N editors. Working Memory: The State of the Science. Oxford, UK: Oxford University Press.

[14] Martin RC Lesch MF Bartha MC. Independence of input and output phonology in word processing and short-term memory. J Mem Lang. (1999) 41:3–29. 10.1006/jmla.1999.2637

[15] Cowan N Morey CC Naveh-Benjamin M. An embedded-processes approach to working memory: how is it distinct from other approaches, and to what ends? In: Logie R, Camos V, Cowan N, editors. Working Memory: The State of the Science. Oxford, UK: Oxford University Press. p. 44–84. Available online at: https://web-b-ebscohost-com.ezproxy.rice.edu/ehost/ebookviewer/ebook/bmxlYmtfXzI2NjE5MTdfX0FO0?sid=08271996-2256-4d9c-96d7-808d09245432@sessionmgr101&vid=0&format=EB&lpid=lp_44&rid=0 (accessed March 12, 2021).

[16] Oberauer K. Design for a working memory. In: Ross BH editor. The Psychology of Learning and Motivation. Elsevier Academic Press (2009). p. 45–100. 10.1016/S0079-7421(09)51002-X

[17] Baddeley A. Working Memory. New York, NY, US: Clarendon Press/Oxford University Press (1986).

[18] Cowan N. The magical number 4 in short-term memory: a reconsideration of mental storage capacity. Behav Brain Sci. (2001) 24:87–114. 10.1017/S0140525X01003922 11515286

[19] McElree B. Working memory and focal attention. J Exp Psychol Learn Mem Cogn. (2001) 27:817–35. 10.1037/0278-7393.27.3.817 11394682 PMC3077110

[20] Martin N Saffran EM. Language and auditory-verbal short-term memory impairments: evidence for common underlying processes. Cogn Neuropsychol. (1997) 14:641–82. 10.1080/026432997381402

[21] Martin RC Breedin SD. Dissociations between speech perception and phonological short-term memory deficits. Cogn Neuropsychol. (1992) 9:509–34 10.1080/02643299208252070

[22] Purcell JJ Turkeltaub PE Eden GF Rapp B. Examining the central and peripheral processes of written word production through meta-analysis. Front Psychol. (2011) 2:239. 10.3389/fpsyg.2011.00239 22013427 PMC3190188

[23] Cipolotti L Bird CM Glasspool DW Shallice T. The impact of deep dysgraphia on graphemic buffer disorders. Neurocase. (2004) 10:405–19. 10.1080/13554790490893995 15788280

[24] Glasspool DW Shallice T Cipolotti L. Towards a unified process model for graphemic buffer disorder and deep dysgraphia. Cogn Neuropsychol. (2006) 23:479–512. 10.1080/02643290500265109 21049341

[25] Sage K Ellis AW. Lexical influences in graphemic buffer disorder. Cogn Neuropsychol. (2004) 21:381–400. 10.1080/02643290342000438 21038212

[26] Ward J Romani C. Serial position effects and lexical activation in spelling: evidence from a single case study. Neurocase. (1998) 4:189–206. 10.1080/13554799808410621

[27] Yue Q Martin RC Hamilton AC Rose NS. Non-perceptual regions in the left inferior parietal lobe support phonological short-term memory: evidence for a buffer account? Cereb Cortex. (2019) 29:1398–413. 10.1093/cercor/bhy037 29522178

[28] Rapp B Dufor O. The neurotopography of written word production: an FMRI investigation of the distribution of sensitivity to length and frequency. J Cogn Neurosci. (2011) 23:4067–81. 10.1162/jocn_a_00109 21812571

[29] Shallice T Vallar G. The impairment of auditory-verbal short-term storage. In: Vallar G Shallice T editors. Neuropsychological Impairments of Short-Term Memory. New York, NY, US: Cambridge University Press. p. 11–53.

[30] Pisoni A Mattavelli G Casarotti A Comi A Riva M Bello L. The neural correlates of auditory-verbal short-term memory: a voxel-based lesion-symptom mapping study on 103 patients after glioma removal. Brain Struct Funct. (2019) 224:2199–211. 10.1007/s00429-019-01902-z 31177297

[31] Paulesu E Frith CD Frackowiak RS. The neural correlates of the verbal component of working memory. Nature. (1993) 362:342–5. 10.1038/362342a0 8455719

[32] Yue Q Martin RC. Maintaining verbal short-term memory representations in non-perceptual parietal regions. Cortex. (2021) 138:72–89. 10.1016/j.cortex.2021.01.020 33677329

[33] Chang EF Edwards E Nagarajan SS Fogelson N Dalal SS Canolty RT. Cortical spatio-temporal dynamics underlying phonological target detection in humans. J Cogn Neurosci. (2011) 23:1437–46. 10.1162/jocn.2010.21466 20465359 PMC3895406

[34] Mesgarani N Cheung C Johnson K Chang EF. Phonetic feature encoding in human superior temporal gyrus. Science. (2014) 343:1006–10. 10.1126/science.1245994 24482117 PMC4350233

[35] Hillis AE Kane A Tuffiash E Beauchamp NJ Barker PB Jacobs MA. Neural substrates of the cognitive processes underlying spelling: evidence from MR diffusion and perfusion imaging. Aphasiology. (2002) 16:425–38. 10.1080/02687030244000248

[36] Rapp B Lipka K. The literate brain: the relationship between spelling and reading. J Cogn Neurosci. (2011) 23:1180–97. 10.1162/jocn.2010.21507 20433242 PMC3106999

[37] Fischer-Baum S. A common representation of serial position in language and memory. In: Federmeier KD Watson DG editors. The Psychology of Learning and Motivation: Current Topics in Language. The Psychology of Learning and Motivation. San Diego, CA, US: Elsevier Academic Press. p. 31–54. 10.1016/bs.plm.2018.08.002

[38] Scolari M Seidl-Rathkopf KN Kastner S. Functions of the human frontoparietal attention network: evidence from neuroimaging. Curr Opin Behav Sci. (2015) 1:32–9. 10.1016/j.cobeha.2014.08.003 27398396 PMC4936532

[39] Wechsler D. Wechsler Memory Scale (WMS-III). San Antonio, TX: Psychological Corporation (1997).

[40] Dunn LM Dunn DM. Peabody Picture Vocabulary Test. 4th ed. Circle Pines, MN: American Guidance Service (2007).

[41] Goodman RA Caramazza A. The Johns Hopkins University Dysgraphia Battery. Baltimore, MD: Johns Hopkins University (1985).

[42] Rossion B Pourtois G. Revisiting Snodgrass and Vanderwart's object pictorial set: the role of surface detail in basic-level object recognition. Perception. (2004) 33:217–36. 10.1068/p5117 15109163

[43] Snodgrass JG Vanderwart M. A standardized set of 260 pictures: norms for name agreement, image agreement, familiarity, and visual complexity. J Exp Psychol. (1980) 6:174–215. 10.1037/0278-7393.6.2.174 7373248

[44] Rorden C Brett M. Stereotaxic display of brain lesions. Behav Neurol. (2000) 12:191–200. 10.1155/2000/421719 11568431

[45] Brant-Zawadzki M Atkinson D Detrick M Bradley WG Scidmore G. Fluid-attenuated inversion recovery (FLAIR) for assessment of cerebral infarction. Initial clinical experience in 50 patients. Stroke. (1996) 27:1187–91. 10.1161/01.STR.27.7.1187 8685926

[46] Neumann AB Jonsdottir KY Mouridsen K Hjort N Gyldensted C Bizzi A. Interrater agreement for final infarct MRI lesion delineation. Stroke. (2009) 40:3768–71. 10.1161/STROKEAHA.108.545368 19797188

[47] Rorden C Bonilha L Fridriksson J Bender B Karnath H-O. Age-specific CT and MRI templates for spatial normalization. Neuroimage. (2012) 61:957–65. 10.1016/j.neuroimage.2012.03.020 22440645 PMC3376197

[48] Nachev P Coulthard E Jäger HR Kennard C Husain M. Enantiomorphic normalization of focally lesioned brains. Neuroimage. (2008) 39:1215–26. 10.1016/j.neuroimage.2007.10.002 18023365 PMC2658465

[49] Desikan RS Ségonne F Fischl B Quinn BT Dickerson BC Blacker D. An automated labeling system for subdividing the human cerebral cortex on MRI scans into gyral based regions of interest. NeuroImage. (2006) 31:968–80. 10.1016/j.neuroimage.2006.01.021 16530430

[50] DeMarco AT Turkeltaub PE. A multivariate lesion symptom mapping toolbox and examination of lesion-volume biases and correction methods in lesion-symptom mapping. Hum Brain Mapp. (2018) 39:4169–82. 10.1002/hbm.24289 29972618 PMC6647024

[51] Zhang Y Kimberg DY Coslett HB Schwartz MF Wang Z. Multivariate lesion-symptom mapping using support vector regression. Hum Brain Mapp. (2014) 35:5861–76. 10.1002/hbm.22590 25044213 PMC4213345

[52] Sato M. The neurobiology of sex differences during language processing in healthy adults: a systematic review and a meta-analysis. Neuropsychologia. (2020) 140:107404. 10.1016/j.neuropsychologia.2020.107404 32087207

[53] Ivanova MV Herron TJ Dronkers NF Baldo JV. An empirical comparison of univariate versus multivariate methods for the analysis of brain–behavior mapping. Hum Brain Mapp. (2021) 42:1070–101. 10.1002/hbm.25278 33216425 PMC7856656

[54] Ivanova MV Dragoy OV Kuptsova SV Akinina YuS Petrushevskii AG Fedina ON. Neural mechanisms of two different verbal working memory tasks: a VLSM study. Neuropsychologia. (2018) 115:25–41. 10.1016/j.neuropsychologia.2018.03.003 29526647 PMC6658104

[55] Mirman D Landrigan J-F Kokolis S Verillo S Ferrara C Pustina D. Corrections for multiple comparisons in voxel-based lesion-symptom mapping. Neuropsychologia. (2018) 115:112–23. 10.1016/j.neuropsychologia.2017.08.025 28847712 PMC5826816

[56] Price D. MNI2FS: High Resolution Surface Rendering of MNI Registered Volumes GitHub. (2021). Available online at: https://github.com/dprice80/mni2fs. (accessed February 26, 2021).

[57] Tamber-Rosenau BJ Marois R. Central attention is serial, but midlevel and peripheral attention are parallel—A hypothesis. Atten Percept Psychophys. (2016) 78:1874–88. 10.3758/s13414-016-1171-y 27388496 PMC5014686

[58] Martin A Chao LL. Semantic memory and the brain: structure and processes. Curr Opin Neurobiol. (2001) 11:194–201. 10.1016/S0959-4388(00)00196-3 11301239

[59] Ralph MAL Jefferies E Patterson K Rogers TT. The neural and computational bases of semantic cognition. Nat Rev Neurosci. (2017) 18:42–55. 10.1038/nrn.2016.150 27881854

[60] Marslen-Wilson WD. Functional parallelism in spoken word-recognition. Cognition. (1987) 25:71–102. 10.1016/0010-0277(87)90005-9 3581730

[61] Baddeley A Gathercole S Papagno C. The phonological loop as a language learning device. Psychol Rev. (1998) 105:158–73. 10.1037/0033-295X.105.1.158 9450375

[62] Freedman ML Martin RC. Dissociable components of short-term memory and their relation to long-term learning. Cogn Neuropsychol. (2001) 18:193–226. 10.1080/02643290126002 20945211

[63] Sala JB Rämä P Courtney SM. Functional topography of a distributed neural system for spatial and nonspatial information maintenance in working memory. Neuropsychologia. (2003) 41:341–56. 10.1016/S0028-3932(02)00166-5 12457759

[64] Randerath J Finkel L Shigaki C Burris J Nanda A Hwang P. Is This Within Reach? Left but not right brain damage affects affordance judgment tendencies. Front Hum Neurosci. (2021) 14:531893. 10.3389/fnhum.2020.531893 33584218 PMC7873490

[65] Kim G Jeong B Choi M Kim W-S Han CE Paik N-J. Neural substrates of subcortical aphasia in subacute stroke: voxel-based lesion symptom mapping study. J Neurol Sci. (2021) 420:117266. 10.1016/j.jns.2020.117266 33341084

