# Corrigendum: Distinct neural substrates support phonological and orthographic working memory: implications for theories of working memory

**DOI:** 10.3389/fneur.2024.1369793

**Published:** 2024-01-29

**Authors:** Jeremy Purcell, Brenda Rapp, Randi C. Martin

**Affiliations:** ^1^Maryland Neuroimaging Center, University of Maryland, College Park, MD, United States; ^2^Cognitive Science Department, Johns Hopkins University, Baltimore, MD, United States; ^3^Department of Psychological Sciences, Rice University, Houston, TX, United States

**Keywords:** working memory, phonological working memory, orthographic working memory, multivariate lesion symptom mapping, working memory deficits, buffer theories, embedded processes theories

In the published article, there was an error in the legend for Figure 4 as published. The color assignments to the different clusters in Figure 4 were incorrect. Phonological Working Memory should be associated with the green color, not the magenta color. Orthographic Working Memory should be associated with the magenta color, not the green color. The corrected legend appears below with the color names correctly assigned to their respective cognitive functions.

SVR-LSM results for Phonological Working Memory (green) and Orthographic Working Memory (magenta). Clusters projected onto a left hemisphere standard MNI152 cortical surface using mni2fs (56). Each image depicts the results using thresholds which, from left to right, are based on increasingly more lenient thresholds from the permutation analysis. Although there is some overlap (white) at the two most lenient thresholds, there is none at the more conservative max and 10th voxel thresholds.

The authors apologize for this error and state that this does not change the scientific conclusions of the article in any way. The original article has been updated.

